# Age-Related Declines in Early Sensory Memory: Identification of Rapid Auditory and Visual Stimulus Sequences

**DOI:** 10.3389/fnagi.2016.00090

**Published:** 2016-05-06

**Authors:** Daniel Fogerty, Larry E. Humes, Thomas A. Busey

**Affiliations:** ^1^Department of Communication Sciences and Disorders, University of South CarolinaColumbia, SC, USA; ^2^Department of Speech and Hearing Sciences, Indiana UniversityBloomington, IN, USA; ^3^Department of Brain and Psychological Sciences, Indiana UniversityBloomington, IN, USA

**Keywords:** temporal processing, sensory memory, aging, psychophysics, cognitive hearing science

## Abstract

Age-related temporal-processing declines of rapidly presented sequences may involve contributions of sensory memory. This study investigated recall for rapidly presented auditory (vowel) and visual (letter) sequences presented at six different stimulus onset asynchronies (SOA) that spanned threshold SOAs for sequence identification. Younger, middle-aged, and older adults participated in all tasks. Results were investigated at both equivalent performance levels (i.e., SOA threshold) and at identical physical stimulus values (i.e., SOAs). For four-item sequences, results demonstrated best performance for the first and last items in the auditory sequences, but only the first item for visual sequences. For two-item sequences, adults identified the second vowel or letter significantly better than the first. Overall, when temporal-order performance was equated for each individual by testing at SOA thresholds, recall accuracy for each position across the age groups was highly similar. These results suggest that modality-specific processing declines of older adults primarily determine temporal-order performance for rapid sequences. However, there is some evidence for a second amodal processing decline in older adults related to early sensory memory for final items in a sequence. This selective deficit was observed particularly for longer sequence lengths and was not accounted for by temporal masking.

## Introduction

Concomitant declines in sensory and cognitive function in older adults are increasingly apparent and guide predominant theories of cognitive aging (see Schneider and Pichora-Fuller, 2000). The examination of the interplay between these different functions of information processing is essential to understanding the relation between sensory and cognitive decline associated with normal healthy aging. This underpins major assumptions of competing theories of sensory and cognitive aging which posit sensory, cognitive, or latent factors as initiating functional decline (Schneider and Pichora-Fuller, 2000; see also Wayne and Johnsrude, 2015). One way of examining this relationship is to dissociate sensory and cognitive contributions to behavioral performance of a single task. Such a method, employed in the current study, defines how age-related declines in either factor contribute to final task performance. In this study, we tested adults on auditory and visual temporal-order (i.e., sequencing) tasks, equated overall performance for the sequence, and examined remaining (i.e., residual) differences in performance due to individual item errors across the sequence. This method allowed us to define the contributions of memory for the serial order and identity of individual items to task performance (i.e., sequence identification) independently of the speed of auditory or visual temporal processing, i.e., the ability to rapidly process stimulus events. Comparison of these trends across modalities allowed us to assess whether any additional memory declines were common across modalities, indicating a common source (or process) of age-related decline, or were modality specific, indicating that sensory changes exert independent influences on observed processing declines with increasing age. Thus, the purpose of this study was to investigate the role of early sensory memory in explaining temporal processing abilities across auditory and visual modalities. This study motivates the investigation of how sensory and cognitive factors are individually recruited by specific task demands and help to explain the source(s) of age-related declines in task performance. This work complements additional work that has investigated individual differences in sensory and cognitive functioning across multiple tasks and modalities (Humes et al., 2013).

A decline in memory performance is one of the more common complaints among older adults. Making use of temporal patterns in the occurrence of items to be recalled, common in memory tasks, can be particularly challenging for older adults (Burke and Light, 1981; Salthouse, 1991; Golomb et al., 2008). Psychophysical temporal-order tasks that test a listener’s ability to rapidly process a sequence of auditory or visual events, such as tones, vowels, or visual symbols, have commonly been used to index perceptual temporal-processing abilities. This measurement is typically done by varying the rate of item presentation to determine how fast the listener can process the sequence with a given level of accuracy (either by determining only the stimulus order or in combination with determining stimulus identity, as implemented here). There is now good evidence of age-related declines for various aspects of temporal processing across modalities (e.g., Moore et al., 1992; Fitzgibbons and Gordon-Salant, 1998; Strouse et al., 1998). However, performance on such tasks is determined not only by the speed of sensory information processing, but by other aspects of that task involving the maintenance of stimulus features, categorization, and retrieval. These abilities involve a short-term sensory memory store that may remain available following the stimulus presentation (Sperling, 1960; Atkinson and Shiffrin, 1968) and facilitate perceptually integrating sequential items (Hogben and di Lollo, 1974; Di Lollo, 1977). Fine grained acoustic cues are coded in auditory short term memory and are essential for discriminating between rapid sequences of vowels (Pisoni, 1973). However, this early encoding of fine acoustic details may be lost in subsequent processing due to processes such as interference of successive sounds and decay over time (Pisoni, 1975), both of which occur in temporal-order tasks. In addition, later categorization processes can lead to a decay in these fine grained representations (e.g., Crowder, 1971). As short-term memory plays an integral role in speech perception (Pisoni, 1975), understanding how well this information is encoded and retained in memory could provide significant insight into speech perception deficits in older adults.

### Temporal Order and Sensory Memory

Our previous investigations have demonstrated that older adults perform significantly poorer (i.e., require larger delays between the onsets of stimuli) than younger adults on auditory temporal-order tasks (Fogerty et al., 2010; Humes et al., 2010). Data analyses from these earlier studies reveal similar item-identification error patterns across younger, middle-aged, and older adults (Fogerty et al., 2012). Thus, when stimuli are sufficiently audible, all age groups appear to make similar types of identification errors indicating that older adults have preserved vowel identification abilities. However, in effortful listening tasks, such as listening in noise or the rapid sequences presented here, memory performance can be impaired due to reduced encoding of the fine-grained stimulus details. This decline in recall may occur even when items are correctly identified during an encoding stage (Rabbitt, 1968, 1991; Pichora-Fuller et al., 1995; Surprenant, 1999, 2007; Kjellberg et al., 2008). While occurring on a very brief time-scale, the reduced temporal processing abilities of older adults may be the result of combined peripheral temporal processing declines and declines in early sensory memory for fine-grained stimulus details. These combined deficits could have a profound impact on speech understanding, which requires processing and ordering rapid acoustic features and segments and recalling them for later interpretation.

At such rapid presentations, the ability to preserve fine-grained acoustic details in memory for short periods of time in order to be able to discriminate between potential stimuli is essential to determining maximum performance. The amount of auditory information available after the stimulus presentation (Crowder and Morton, 1969; Fujisaki and Kawashima, 1970; Pisoni, 1973; Darwin and Baddeley, 1974) or the similarity of the observed stimulus to stored representations (Nairne, 1990) determines the ability to discriminate and ultimately recall stimulus events (Surprenant and Neath, 1996).

Classic studies of early sensory memory have demonstrated that when auditory stimuli are presented, listeners recall the first and last items in a sequence the best, termed the primacy and recency effect, respectively (Crowder, 1976). However, the recency effect is not observed for visual presentations (LeCompte, 1992; see also Penney, 1989) as well as transient, categorically perceived auditory stimuli, such as stop consonants (Cole, 1973; Pisoni, 1973). Such evidence has been interpreted as reflecting that final items indicate a precategorical (Crowder and Morton, 1969) or fine-grained memory trace of the individual stimulus features (Fujisaki and Kawashima, 1970; Pisoni, 1973, 1975). Thus, recall of final items in a sequence reflect the detailed encoding of stimulus features, while recall of earlier items in the sequence are more impacted by other processes, such as interference of the sensory memory trace by later items (Pisoni, 1975). Therefore, examining recall performance across the stimulus sequence can help localize an underlying deficit in sensory memory that may be responsible for the poorer performance of older adults on temporal order tasks.

The current analysis was conducted on a set of temporal-order tasks, involving two- and four-item vowel and letter sequences, from earlier reports (Busey et al., 2010; Fogerty et al., 2010; Humes et al., 2010, 2013) that have documented age-related declines in temporal order processing for these same stimuli. The purpose of this analysis was to determine how temporal order abilities might be associated with early processes of sensory memory. Evidence from standard measures of working memory has suggested poorer item recall for older adults across serial positions (e.g., Fiore et al., 2012). The current analysis investigated identification accuracy as a function of vowel position, sequence length, rate of presentation, and modality.

### Declines in Temporal Order with Increasing Age

The psychophysical perception of temporal order is essential for rapidly decoding information within a sequence of events. Undoubtedly, this temporal ability plays a large role in processing rapid speech events, facilitated by dynamic frequency changes across the sequence (Dorman et al., 1975). Age-related temporal-order deficits are likely to underlie some of the speech understanding difficulties that older listeners face (Trainor and Trehub, 1989). While audibility is the primary predictor of age-related auditory processing, Humes and Christopherson (1991) found that the four auditory perception tasks that discriminated between the speech-understanding performance of younger and older listeners had a temporal component (i.e., embedded test-tone task, temporal-order discrimination for tones and syllables tasks, and mid-frequency pure-tone discrimination task possibly related to a timing-based mechanism), two of which involved temporal ordering. While auditory temporal processing for simple patterns is sometimes preserved for older listeners, such processing generally breaks down at faster processing rates and for more complex patterns (Fitzgibbons and Gordon-Salant, 1998). With increased task and stimulus complexity, additional cognitive demands are placed upon the listener (Pichora-Fuller, 2003). Thus, declines in attention and memory that are also associated with age-related declines may also play a role in decreased processing capacity of older listeners (Schneider et al., 2010).

The reason for this impairment in temporal-order processing is not entirely clear. Fitzgibbons and Gordon-Salant (1998) initially suggested an auditory processing-rate limitation independent of cognitive mechanisms as the underlying cause. Recent evidence has suggested modality-specific processes underlying temporal processing across a large number of tasks (Humes et al., 2009). However, there may also be a more general difficulty, not attributed to a sensory/perceptual level (Fitzgibbons et al., 2006).

In order to investigate the physiological bases of temporal-order processing, Lewandowska et al. (2008) measured auditory evoked potentials and found increased amplitudes in N1 and P2 components at shorter inter-stimulus intervals (ISIs) for older participants. These results demonstrate impaired temporal-order processing for older adults at higher central auditory levels (Szelag et al., 2009). Additional neural evidence comes from investigations exploring the mismatch negativity (MMN) response. The MMN is generated by a sensory-memory based comparison between an actual stimulus and a prediction based on previous stimulation (Schröger, 2005) and can index automatic processing of temporal order in auditory sensory memory (e.g., Bendixen and Schröger, 2008). Rimmele et al. (2012) measured MMN responses to brief two-tone sequences and found reduced MMN amplitude for older adults, indicative of reduced sensory memory with increasing age for auditory temporal order processing. This reflects a more automatic processing of the temporal structure in sensory memory (Näätänen et al., 2010) that does not involve explicit memory or attention related deficits at higher levels of processing (Rimmele et al., 2015). Indeed, reduced MMN response at rapid rates (i.e., short ISIs) is a robust feature of aging (reviewed by Cheng et al., 2013). Thus, there is increasing evidence for the involvement of sensory memory in perceptual declines of temporal-order with increasing age, in addition to a perceptual temporal slowing. However, much of this evidence comes from passive MMN tasks. The current investigation provides an analysis of identification errors across rapid stimulus sequences for auditory and visual presentations.

### Examining the Association between Sensory and Cognitive Decline

As stated earlier, a number of theories have now been outlined to explain observed declines in sensory and cognitive function with increasing age (Schneider and Pichora-Fuller, 2000). From the foregoing, it is difficult to discern the relative contributions of sensory-specific declines in the auditory periphery and general cognitive declines. One behavioral method to examine such cognitive contributions to listener performance is to examine correlations between performance on independent tasks of auditory and cognitive processing. In support of this relationship, Fogerty et al. (2010) found that cognitive abilities, as indexed by the Wechsler Adult Intelligence Scale-III (WAIS-III; Wechsler, 1997), accounted for between 8% and 29% of the variance on several different temporal-order tasks among older adult listeners. The best prediction provided by cognitive abilities occurred for monaural and dichotic auditory temporal order tasks in which listeners had to identify two vowels presented in a rapid sequence. In addition, structural equation modeling based on a large test battery of sensory and cognitive abilities has suggested that age-related changes in global cognitive function are mediated by global changes in sensory processing (Humes et al., 2013), justifying a detailed investigation of how this sensory-cognitive link operates for individual task performance.

A second method of examining cognitive contributions to temporal processing is to examine performance on the auditory temporal-order task itself. This type of statistical examination can determine if there are differences between younger and older adults in the accuracy patterns for individual items in the sequence that remain after accounting for differences in temporal processing thresholds. The current analysis extends the previous investigation (Fogerty et al., 2010) by examining the accuracy for individual items across the sequence with a larger set of participants across age groups. Such an analysis can help to determine perceptual dependencies between sequence positions and the role of sensory memory during serial item recall. Many previous studies have documented age-related declines in auditory temporal processing. However, the current exploration describes the type of difficulties that older adults have in processing stimulus sequences. These difficulties are defined here according to performance differences across participants that are observed at the various positions in a temporal sequence.

Finally, a central question related to auditory temporal processing declines is whether they are specific to the auditory modality, or reflect more general, amodal declines in processing that likely occur at more central levels of processing or otherwise reflect systemic changes in neural function. Examining performance for similar tasks across modalities is one way of identifying modality-specific auditory processing deficits (e.g., McFarland and Cacace, 1995; Cacace and McFarland, 1998; Humes et al., 2009). If differences in recalling item order are independent between the different modalities, then sensory-specific declines may underlie older adult performance. However, the degree to which temporal order performance results in similar types of recall errors for auditory and visual sequences would suggest the possible involvement of a common underlying factor (i.e., a common cause, Lindenberger et al., 2001); although conceivably, similar perceptual errors could occur across modalities as the result of different sensory-specific processes. Toward this end, the current investigation also explored visual temporal order performance on a set of temporal order measures that were closely matched to the methods used for the auditory tasks (see Busey et al., 2010, for a discussion of visual threshold performance). Previous investigations of auditory and visual stimuli, matched for difficulty and stimulus features, demonstrate similar representations in short term memory for the two modalities (Visscher et al., 2007), suggesting the potential for any age-related declines in sensory memory to be observed across modality. Such an investigation may provide further evidence of whether observations of age-related temporal-order declines are the result of more general limitations that explain performance variance across task modality, in addition to contributions from sensory-specific auditory temporal processing deficits.

## Experiment 1: Monaural Auditory Sequences

### Listeners

**Figure 1:**
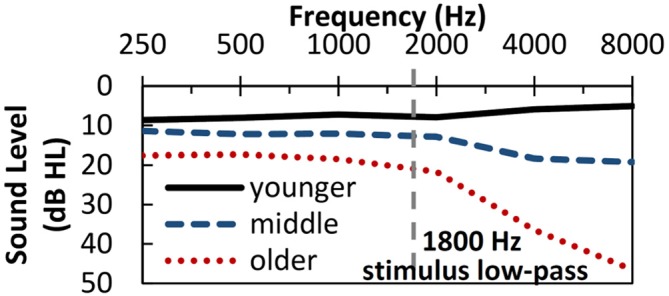
**Mean audiogram for listeners in each of the three age groups in the test ear (right).** Dashed vertical line represents low-pass filter cut-off frequency applied to the vowel stimuli.

Three groups of listeners participated in the current experiment: younger adult listeners [*N* = 78, Age: *M* = 23 years (18–31 years)]; middle-aged adult listeners [*N* = 40, Age: *M* = 48 years (40–55 years)]; and older adult listeners [*N* = 141, Age: *M* = 70 years (60–87 years)]. Screening procedures required each listener to identify vowel and letter stimuli in isolation with at least 90% correct accuracy and pass the Mini-Mental State Exam (MMSE; Folstein et al., 1975). All listeners completed baseline measures of auditory sensitivity (i.e., audiometric thresholds), visual acuity (i.e., Snellen chart) and were required to have no evidence of middle ear pathology (air-bone gaps <10 dB and normal tympanograms). Figure 1 displays the mean audiometric data in the test ear for the three groups of listeners. All age groups had nearly identical average audiometric thresholds for the two ears. As hearing sensitivity declines with age (Pearson et al., 1995), older listeners had expectedly elevated audiometric thresholds compared to the other groups. Stimuli were specifically designed to ensure sufficient audibility for all listeners.

### Stimuli

Stimuli consisted of four vowels recorded by a male talker rapidly saying the words “pot, pet, pit, put” in a carrier phrase, “The first word is ___ now”. A single production of each word was digitally edited to remove all voiceless sounds, leaving only the central voiced pitch periods. As these were naturally produced vowels, some information about the neighboring consonants likely remained. Vowel stimuli were modified in STRAIGHT (Kawahara et al., 1999), a speech synthesis and analysis program, to be 70-ms long and have a constant fundamental frequency at 100 Hz. Modified stimuli were low-pass filtered at 1800 Hz to reduce effects of high-frequency hearing loss and root-mean-square (RMS) normalized. Selection of vowel productions ensured that the first two formants were preserved below 1800 Hz, the upper cutoff frequency of the stimuli. Stimuli were presented at 83 dB sound pressure level (SPL) via an ER-3A insert earphone in a sound attenuating booth using Tucker Davis Technologies System III hardware.

### Design and Procedures

All listeners completed testing for two- and four-item sequence lengths rapidly presented to the test ear (usually the right ear, 98.6% of cases). The delay for subsequent items in each sequence was manipulated to determine the delay corresponding to a given identification-performance criterion. This is referred to as the stimulus onset asynchrony (SOA). In these tasks the vowels were allowed to temporally overlap each other, but not occur simultaneously. In practice, all age groups were able to complete the two-item task with criterion SOAs yielding some temporal overlap of the vowels. In contrast, for the four-item sequences, all age groups required SOAs that produced a temporal delay (i.e., silence) between presentations of the vowels, and thus, were not overlapping for this task. For four-item sequences, vowels were allowed to repeat during a trial, but not within adjacent sequence positions. All tasks were preceded by familiarization tasks with feedback. Listeners used a touch screen monitor to respond by pressing large buttons labeled according to the vowels that they heard presented on a given trial. Columns of these four buttons were provided, one column for each item presented. Listeners were required to identify the entire sequence by selecting one vowel per column in the serial order of their presentation.

Overall performance on this task was defined by correct identification of the serial order for entire sequence. As all possible combinations of the four vowels were tested, a total of 12 different sequences were possible for the two-item task (chance performance for sequence identification was 8.3%), with chance performance near zero for the large combination of sequence possibilities for the four-item task.

To obtain psychometric thresholds, the method of constant stimuli was used to present stimulus sequences over a fixed range of six SOA values. Due to the large variability between listeners, testing was completed using a two-step procedure for both experimental tasks. First, a *wide-range* test block was used to provide an initial estimate of an individual’s SOA threshold. Fixed parameters over a wide SOA range were used: six SOA steps spanning 10–135 ms for the 2-item and 35–160 ms (or 85–210 ms for older listeners) for the four-item tasks. Second, a set of three *narrow-range* test blocks were used to provide a more precise measure of each individual’s SOA threshold. Variable parameters for narrow-range testing were estimated from each individual’s wide-range block. Listeners completed three blocks using a small step size with a range centered at the SOA threshold estimated from the wide-range block (i.e., the estimated SOA fell approximately midway between the narrow range SOAs tested). SOA thresholds for identifying the entire sequence of vowels were calculated at 50% using a single psychometric function (i.e., Weibull) fit to the pooled data over all three of an individual’s narrow-range blocks. The sequence position analyses conducted here were investigated across the six narrow-range SOAs tested for each listener, centered at their individual threshold, as well as sequence identification performance at the tested SOA nearest their individual threshold.

### Results and Discussion

#### Vowel Identification for Two-Item Sequences

Two sets of data analyses were conducted. First, the effect of vowel position (i.e., the order of the vowel in the sequence: first or second) was investigated at each individual’s threshold for the task. In this way, performance was equated according to each individual’s temporal processing ability. This data was a subset of a second analysis that investigated performance across the six SOAs tested. This second analysis allowed for the investigation of how sequence presentation rate may have impacted position accuracy. As there was high variability across listeners, particularly the older group, non-parametric statistics were used here and in subsequent experiments. This allowed the inclusion of outlier data from listeners who had significant difficulty with the tasks and that we believe represented meaningful variation in abilities that was important to include in the analysis. Therefore, analysis was conducted on median values that were more representative of group performance.

##### Performance at Threshold

**Figure 2:**
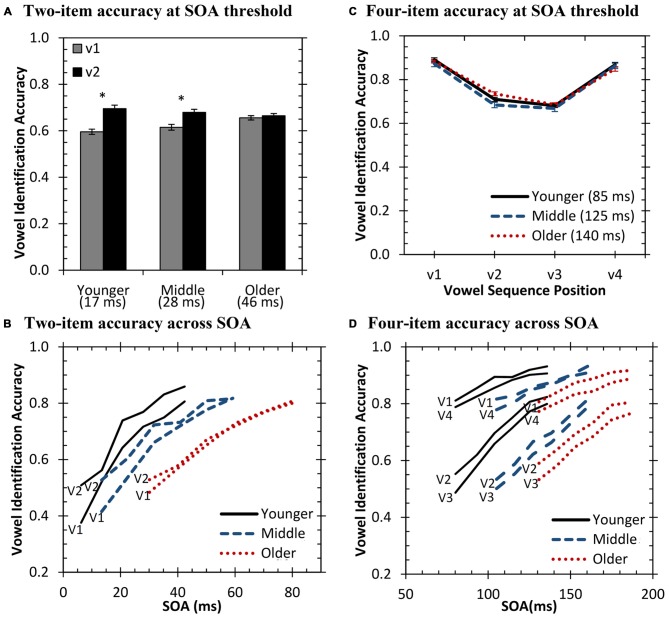
**Auditory vowel identification for two-item (A,B) and four-item (C,D) sequences.** Solid = younger listeners, dash = middle-aged listeners, dotted = older listeners. Median stimulus onset asynchronies (SOA) thresholds for each group are displayed in parentheses **(A,C)**. *p < 0.05.

The non-parametric Kruskal-Wallis test was computed to examine the effect of age group for identifying vowels in either the first or second position in the sequence. Results demonstrated significant group differences for the first sequence position [*H*_(2)_ = 16.5, *p* < 0.001]. Follow-up independent-samples Mann-Whitney U tests demonstrated that younger [*U* = 3206.5, *Z* = −3.8, *p* < 0.001] and middle-aged [*U* = 2915.0, *Z* = −2.7, *p* < 0.01] listeners were less accurate on the first position compared to the older listeners. Figure 2A displays the mean performance of the age groups for each vowel position in the sequence. No significant difference was obtained between the younger and middle-aged groups (*p* > 0.05). As overall performance was equated, this indicates that younger and middle-aged listeners had a greater effect of vowel position compared to the older listeners. However, while it appears that older listeners were more accurate at threshold for identifying the first vowel in the sequence compared to the other age groups, this occurs at a much higher SOA value than the other two groups.

The results of this analysis suggest that older listeners may have diminished processing for two-item sequences relative to younger and middle-aged listeners. The greater accuracy of the final vowel by the latter two groups (younger: Wilcoxon *Z* = −5.2, *p* < 0.001; middle-aged: Wilcoxon *Z* = −3.4, *p* < 0.001) suggests greater perceptual specification of the end of the second vowel (as the vowels were overlapping). This may reflect a better early sensory memory store for fine-grained acoustic details by these two groups. In contrast, the older listeners may have attempted to process the entire sequence holistically or categorically, rather than individually process the extremely short and rapid acoustic cues of the individual vowels. Furthermore, it may be expected that the processing time allowed for processing the sequence would mediate these effects. Therefore, this analysis was also conducted across SOA values tested.

##### Performance Across SOA

Performance was also investigated as a function of the SOA, or sequence presentation rate (i.e., shorter SOAs led to faster presentation rates of the sequence). Figure 2B displays the accuracy of the first and second vowel in the sequence for the three groups at the average SOAs tested for that group. As can be observed, the difference between the groups for this position effect remained robust across SOA values tested.

**Figure 3:**
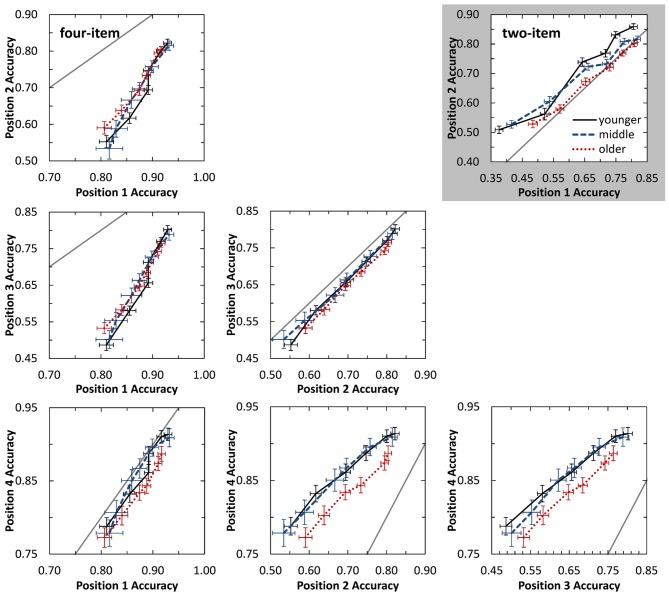
**State-trace plots displaying auditory identification accuracy for one sequence position against the accuracy for a different sequence position.** The gray box displays performance for the two-item sequence, while white plots display performance for all position comparisons on the four-item task. Performance is plotted for the six different SOA values tested, with poorer performance obtained for shorter SOA values. The gray diagonal line plots equivalent performance for the two sequence positions. Performance below this line indicates better performance for earlier occurring items, while performance above the line indicates better performance for more later occurring items. Error bars indicate standard error of the mean.

Observable from Figure 2B, all listener groups demonstrated improved accuracy with increasing SOA. The younger and middle-aged groups showed parallel trends with greater SOAs for middle-aged listeners likely due to beginning declines in temporal processing. Older listeners appear to have a different function, possibly indicating less benefit to vowel identification from equal increases in temporal delay compared to the other two age groups. To investigate this possibility, a state-trace analysis (Bamber, 1979; Prince et al., 2012) was conducted to compare performance between the two sequence positions at fixed levels of accuracy. Based on this inter-item comparison for each subject, the logic of the state-trace analysis suggests that lower performance on one item compared to another item would indicate a deficit, or greater processing required, for the item with lower performance. While comparisons in Figure 2 are dependent on the overall level of performance as controlled for by testing at SOA values determined by individual thresholds, the state-trace analysis demonstrates item performance relative to their own performance at a different sequence position (i.e., if one group of participants is specifically disadvantaged on one particular position). This analysis is better able to characterize mechanistic differences between age group processing across the sequence positions. The gray, highlighted panel in Figure 3 displays this analysis for two-item sequences, which plots accuracy for position 1 against accuracy for position 2. Each point of the curve is performance for the two sequence positions at a single SOA value. Implicit in this analysis is the effect of SOA, with greater SOA values associated with better accuracy. Visual inspection demonstrates that for a given fixed accuracy level for position 1, older listeners demonstrate lower position 2 accuracy. Therefore, across all SOAs tested, older listeners perform more poorly at the final sequence position relative to their performance on the first item compared to the other age groups. By the logic of state trace analysis, we conclude that they are selectively disadvantaged at the final position, and this is independent of their overall levels of accuracy.

While younger listeners significantly identified the second vowel in the sequence better than the first vowel across all SOAs tested (*p* < 0.05), older listeners always identified the two sequence positions equally well. In contrast to the younger and older listeners, middle-aged listeners demonstrated a significant difference between vowel position only at the shortest three SOAs tested (*p* < 0.05). Thus, performance for middle-aged listeners is similar to younger listener performance at short SOAs and older listener performance at the longer SOAs (see also Figure 3). This appears to be reflective of an age-related change in temporal sequence processing, not an effect of SOA, as the position effect for younger and older listeners is consistent across SOAs, even when tested over the same SOA range (~30–50 ms delay).

A number of different reasons could account for the observed age group differences. These results may indicate a difference in listening strategy, rather than actual processing differences. Younger listeners may focus at the end of the sequence, while older listeners may focus on cues of the combined sequence. Middle-age listeners may change strategy based upon the presentation rate. At least for discrimination, it does appear that listeners may sometimes process auditory sequences of vowels, tones, or noises as a single holistic unit, rather than a sequential presentation of independent items (Warren and Bashford, 1993), with listeners recognizing individual items at longer stimulus durations (e.g., Warren and Ackroff, 1976). It may be that the older listeners in the current study were more prone to using holistic processing strategies for sequence identification due to possible difficulty temporally resolving independent items at rapid rates. This may also result from a second possible cause. The results are also consistent with short-term sensory memory impairment in processing final items (Figure 3; Frankish, 2008). As final items result in persistence of the stimulus in sensory memory that underlies perceptual improvements in identifying final items (Cowan, 1984), the relatively poorer final item performance for older adults suggests a rapidly decaying memory trace for this final item. This is in addition to temporal processing declines evidenced by elevated SOA thresholds. As such, older listeners may have a reduced precategorical short term auditory store for the fine-grained acoustic details that may have reduced performance on the final item (Crowder and Morton, 1969; Fujisaki and Kawashima, 1970; Pisoni, 1973; Darwin and Baddeley, 1974; Surprenant and Neath, 1996).

##### Vowel Identification for Four-Item Sequences

Listeners also completed vowel identification for four-item sequences. Unlike the two-item sequences that allowed temporal overlap between the vowels, all listeners required some degree of temporal separation between the vowels to accurately complete the four-item sequences. As before, performance for the four-item sequences was completed in two sets of analyses investigating performance on a subset of the data at each individual’s SOA threshold for identifying the entire sequence with 50% accuracy and investigating performance as a function of the presentation rate (i.e., SOA) across the entire dataset.

##### Performance at Threshold

Performance at threshold was investigated by comparing the serial position curves for the three age groups (displayed in Figure 2C). Results demonstrated that at threshold there was highly similar performance between the three groups. As can be observed from Figure 2C, all listener groups demonstrated a strong primacy and recency effect, which is surprising given these very rapid and short sequences. The non-parametric Kruskal-Wallis test was used to investigate group differences across the four different sequence positions. Results demonstrated a significant difference between the groups at the second sequence position [*H*_(2)_ = 12.3, *p* < 0.01]. *Post hoc* Mann-Whitney U tests demonstrated that older listeners identified the vowel in second position better than younger [*U* = 2734.0, *Z* = −2.2, *p* < 0.05] and middle-aged [*U* = 1204.5, *Z* = −3.3, *p* < 0.001] listeners.

Again, as overall performance across the entire sequence was equated at each listener’s threshold, this difference between the groups indicates that the older listeners have a different relative difference in identification accuracy between the positions in the sequence, most notably for the second sequence position. However, overall, when sequences are fully audible and equated for temporal processing differences, performance among the three age groups is highly similar.

##### Performance Across SOA

Results were also investigated across presentation rates. Figure 2D displays vowel identification across SOA for the four sequence positions. As with the two-item sequences, middle-aged listeners showed a nearly identical function shifted to larger SOAs compared to the younger listeners. This may indicate similar identification and memory processes between these two groups, with declines in temporal processing responsible for the shift. This hypothesis is supported by the statistically similar serial-position curves obtained at each individual’s threshold for the younger and middle-aged groups. In contrast, the older listeners again demonstrated a different function. While older listeners did have better identification of the second vowel in the sequence, they were tested at higher SOA values to equate performance. A state-trace analysis (see white panels in Figure 3) was again conducted on these data for the four-item task to explore differences in accuracy for the different sequence positions. For most of the sequence position comparisons, all three age groups performed similarly. However, a marked difference for older listener performance is observed for several comparisons with the final sequence position (i.e., position 4; see bottom row in Figure 3). In these cases, for a fixed level of performance on position 2 or 3 (e.g., 0.70), performance on position 4 is systematically lower (i.e., relative to performance at other sequence positions) for the older listeners than for other age groups. Combined with the similar results from two-item sequences (gray panel in Figure 3), older listeners appear to display a general, “final item” deficit relative to the advantage the other two groups have for final item identification. Note that it is not the older listeners cannot identify final items—they can—but that their performance on final items does not reach that of younger and middle-aged listeners relative to their performance across the initial portion of the sequence. These results for the older listeners (i.e., red dotted line in Figure 3) suggest that in addition to temporal processing declines, they may also demonstrate involvement of a second mechanism (e.g., sensory memory), as temporal processing and hearing sensitivity were explicitly controlled in this experiment but appear inadequate in explaining this deficit. Experiment 2 examines potential amodal contributions of this secondary factor more fully by testing performance on visual sequences.

##### Investigating Potential Sensory Contributions to Final Item Differences: Temporal Masking

One possible sensory limitation that could underlie differences in processing final items is temporal masking. As backward temporal masking generally leads to more elevated thresholds compared to forward masking (see Humes et al., 2010), reduced accuracy in final position items for older listeners would suggest greater contributions of forward masking from the preceding stimulus. Therefore, we would expect to find larger forward masking deficits in the older listeners. The temporal masking data from Humes et al. (2010) was used to investigate this possibility. In one of the conditions presented in that study, listeners were presented with a vowel babble masker (four staggered, but overlapping repetitions of each of the four stimulus vowels). This masker was presented at 4 dB signal-to-noise ratio (SNR) in both forward and backward masking conditions. The target vowel was allowed to overlap in time with the pattern masker, similarly to the temporal overlap allowed in the temporal order tasks reported here. The SOA threshold was measured adaptively for identifying the target vowel.

Indeed, for this group of listeners, threshold performance for two-item identification was very similar to backward masking thresholds using a vowel-babble masker (i.e., median values within 7-ms across age groups; 49.1 ms for two-item temporal-order compared to 42.6 ms during backward masking for older listeners), while forward masking thresholds were half as large (e.g., 21.7 ms for older listeners). This comparison underscores that two-item SOA values were tested within a range at which temporal masking properties could influence performance. Furthermore, for both younger and older adults, forward and backward masking thresholds were significantly associated with two-item temporal order thresholds (Spearman’s rho: 0.46–0.65, *p* < 0.01). However, a number of sources of evidence argue against a temporal masking explanation of processing differences between the age groups for final items when compared at performance levels that equated these differences in temporal order thresholds across groups, and potentially also associated effects of temporal masking.

First, younger listeners, and to some extent middle-aged listeners, demonstrated consistently better performance for final items across all SOAs tested. However, reduced differences would be expected at longer SOAs due to a release from temporal masking. Second, a temporal masking interpretation would predict elevated forward masking thresholds for older listeners compared to younger listeners. However, Cohen’s effect sizes demonstrated much larger age group differences for the backward masking task (Younger: *N* = 85, *M* = 21.2 ms, *SD* = 19.0; Older: *N* = 136, *M* = 51.1, *SD* = 36.8; *U* = 2083.0, *Z* = −7.8, *d* = 1.07) compared to forward masking (Younger: *M* = 18.1 ms, *SD* = 41.0; Older: *M* = 55.0, *SD* = 55.0; *U* = 2413.5, *Z* = −7.3, *d* = 0.55). Third, the relative difference between forward masking and backward masking was also examined, as this effect might also suggest elevated forward masking thresholds relative to backward masking. No significant difference was obtained between the younger and older listeners (*U* = 4735.0, *Z* = −1.8, *p* > 0.05). This indicates that these two types of temporal masking would have exerted similar effects on both age groups. Combined, these three findings suggest that age-related changes in temporal masking, specific to the sensory modality, cannot fully account for deficits in processing final items by the older listeners. Instead, a sensory memory account of the findings appears most consistent with the data. Experiment 2 was conducted to determine if these differences in identification across the stimulus sequence also occur in the visual modality. Such a finding would suggest an amodal component to age-related changes in temporal order processing.

## Experiment 2: Visual Sequences

Experiment 1 demonstrated remarkable similarity between age groups for the different experimental conditions, with differences for older listeners isolated to final item performance. As mentioned previously, comparison of performance across different modalities is a method that can localize processing differences to either unimodal auditory specific mechanisms or more general amodal mechanisms that operate across sensory modalities. In the current study, a subset of listeners from Experiment 1 was tested with similar methods using visual stimuli. Threshold performance on these visual measures have been discussed in detail previously (see Humes et al., 2009, 2013; Busey et al., 2010). The current analysis of the data reflects performance for individual items in the sequence, which has not been investigated previously to examine similarities in performance for visual and auditory sequences.

### Methods

A subset of the participants who completed the first experiments participated in Experiment 2 (younger: *N* = 49, mean age = 22; middle-age: *N* = 31, mean age = 48; older: *N* = 91, mean age = 71).

Stimuli used in the experiment were the letters M, P, O, and T rendered in 12 point Times font (previously used by Busey et al., 2010). Letters represented approximately equal overlap as defined by a computer search quantifying overlapping pixels between letter pairs. Letters were embedded in a background patch at luminance values slightly higher than the background, subtended 1.16° of visual angle at their widest extents, and were presented to a single visual field location. Stimuli were presented for 30 ms and were separated by one of six SOA values, as with the auditory testing. Note that SOA values less than 30 ms resulted in an overlapping display of the letters, but did not result in changes in brightness of overlapping pixels. SOA thresholds were obtained in the same way as the auditory tasks using a two-step procedure in which a single wide-range of SOA values was first used to estimate performance followed by three subsequent blocks using a narrow range of SOA values. For visual testing, test SOA values were re-estimated for each block, resulting in some differences across blocks (The average standard deviation across SOA test ranges was 6.8 ms for two-item sequences and 19.4 ms for four-item sequences). As with the auditory testing, thresholds were computed using a single psychometric function (i.e., Weibull) fit to the pooled data from these three test blocks. Each listener was tested on a total of 288 trials (3 blocks × 6 SOAs × 16 trials).

For the current analysis, item accuracy was calculated at the test SOA nearest the estimated SOA threshold from the Weibull function. Participants were only included in this analysis if their estimated thresholds were within the test SOA range spanned across the three test blocks, allowing for one standard deviation of variance between the test blocks (for example, a threshold of 5.6 ms was accepted for a test range of 7–49 ms as the variance between the three test blocks for this individual was 1.3 ms). In practice, only nine participants in the two-item condition and none in the four-item condition were included that had thresholds outside of the SOA test range (by an average of 5 ms). However, across three test blocks they still demonstrated reliable thresholds and were included in the analysis. Performance at the test SOA nearest the SOA threshold was examined separately for each block and pooled for final analysis. This is similar to the analysis conducted for Figures 2A,C for auditory testing.

Performance was also calculated across SOA. In this analysis, data were pooled across the three blocks according to the SOA test interval of that block (e.g., the three shortest SOA values across blocks were pooled for calculating performance for the first of six SOA test values). Again, as the same SOA values were not tested across blocks, there was some variability in the actual SOA values that were pooled for analysis for a given listener. On average, the standard deviation across SOA values for the three blocks deviated an average of 19% from the mean SOA across blocks for the two-item task and 13% for the four-item task. This deviation was consistent for each of the six SOA values tested per participant and represents intrinsic within-participant variability in performance. These averaged SOA values can be viewed as a more stable estimate of individual performance, and reliable psychometric functions were obtained over the pooled data.

### Results and Discussion

#### Letter Identification for Two-Item Sequences

**Figure 4:**
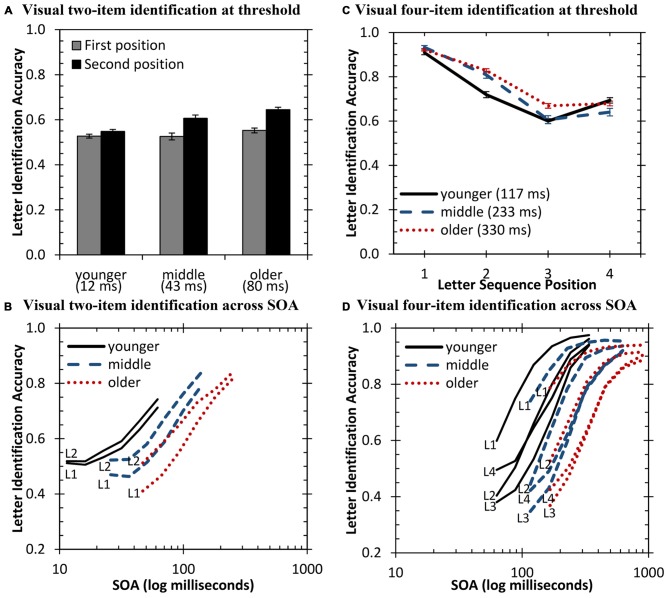
**Visual sequence identification for the three age groups. (A,B)** Visual two-item letter identification. **(C,D)** Visual four-item letter identification across the four sequence positions. Median SOA thresholds for each group are displayed in parentheses **(A,C)**.

The non-parametric Kruskal-Wallis test was computed to examine the effect of age group on letter identification for the two sequence positions. Results demonstrated significant group differences for the second sequence position [*H*_(2)_ = 29.9, *p* < 0.001]. Follow-up independent-samples Mann-Whitney U tests demonstrated significant differences for all group comparisons for the second sequence position with better performance for increasing age group (younger vs. older: *U* = 875.0, *Z* = −5.2, *p* < 0.001; younger vs. middle-aged: *U* = 385.0, *Z* = −.3.2, *p* < 0.001; middle-aged vs. older: *U* = 955.5, *Z* = −2.0, *p* = 0.04). Figure 4 displays the mean performance of the age groups for each vowel position in the sequence. Figure 4A displays this performance data calculated at each individual’s SOA threshold. Performance is also displayed as a function of SOA in Figure 4B.

First, while age-group differences were most apparent for the first sequence position for auditory presentations, the second position resulted in the greatest differences for visual presentations. Second, differences between the relative accuracy of the first and second position are also apparent. For auditory presentations, younger adults demonstrated position asymmetry (i.e., better final item identification), while older adults identified both positions similarly. Again, the opposite was true for visual presentations. The older adults identified the second item more accurately (9 percentage-point difference, *Z* = −6.2, *p* < 0.001, Cohen’s *d* = 0.91), while the younger adults had minimal difference between the two positions (2 percentage-point difference, *Z* = −3.4, *p* = 0.001, Cohen’s *d* = 0.33). This observation is also consistent with visual inspection of Figure 4B, which plots performance across SOA. These differences in item accuracy suggest that sensory-specific processing is more likely responsible for individual error response patterns rather than amodal processing. This result further supports previously reported data on temporal processing in these same participants over a host of other temporal processing tasks suggesting separate sensory declines in performance (see Humes et al., 2009).

**Figure 5:**
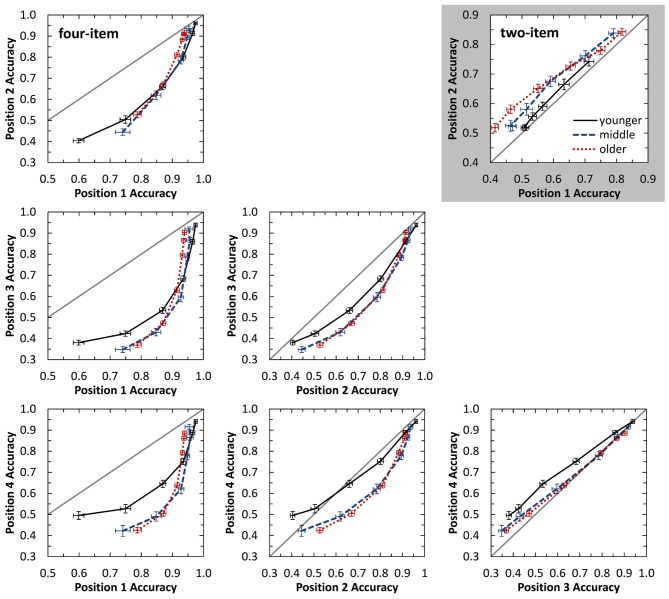
**State-trace plots displaying visual identification accuracy for one sequence position against the accuracy for a different sequence position.** The gray box displays performance for the two-item sequence, while white plots display performance for all position comparisons on the four-item task. Performance is plotted for the six different SOA values tested, with poorer performance obtained for shorter SOA values. The gray diagonal line plots equivalent performance for the two sequence positions. Performance below this line indicates better performance for earlier occurring items, while performance above the line indicates better performance for more later occurring items. Error bars indicate standard error of the mean.

A state-trace analysis was also conducted for the two-item visual data and is displayed as the grayed panel in Figure 5. Results from this analysis are consistent with the observations listed above. Younger adults demonstrated little difference in accuracy between item positions, while middle-aged and older adults demonstrated a benefit for items in final position across SOA for these visual two-item sequences. This represents an exception to the final position deficit of older adults that was found in Experiment 1 with auditory two- and four-item sequences. However, it is important to note that even with this performance advantage, older adults still performed significantly poorer at identifying the entire sequence.

#### Letter Identification for Four-Item Sequences

For four-item sequences, the non-parametric Kruskal-Wallis test was again used to investigate group differences across the four different sequence positions (see Figure 4C). Results demonstrated a significant difference between the groups at the second [*H*_(2)_ = 39.3, *p* < 0.001] and third sequence position [*H*_(2)_ = 17.3, *p* < 0.001]. *Post hoc* Bonferroni-adjusted Mann-Whitney U tests demonstrated lower accuracy for younger adults at the second position [middle-aged: *U* = 348.0, *Z* = 3.9, *p* < 0.001; older: *U* = 727.5, *Z* = 6.1, *p* < 0.001] and third sequence position [middle-aged: *p* > 0.05; older: *U* = 1223.5, *Z* = 3.8, *p* < 0.001]. Performance between middle-aged and older adults was significantly different at the third sequence position (*U* = 817.5, *Z* = 2.8, *p* < 0.01). However, overall, when sequences are equated for visual temporal processing differences, performance among the three age groups is highly similar, at least when examining performance at each individual’s threshold.

Performance was also examined as a function of SOA over the entire dataset. Visual inspection of these data in Figure 4D demonstrates a clear rank ordering of the participant groups for each item position, with younger adults consistently performing better at smaller SOA values than middle-aged adults, who consistently performed better than older adults.

The difference between groups may be related to differences processing more recent stimuli. To investigate this, accuracy between the third and fourth positions was compared using Wilcoxon Signed Ranks tests. Younger adults performed significantly better in the last position (*Z* = −4.8, *p* < 0.001); whereas, older adults performed no differently (*p* > 0.05). Performance for middle-aged adults reached marginal significance (*Z* = −2.0, *p* = 0.04). This indicates that older adults, and possibly some middle-aged adults, have a more rapidly decaying representation of the stimulus in sensory memory for items at the end of the sequence.

The possibility of a decaying sensory memory trace for final items was investigated again using a state-trace analysis and is displayed in Figure 5. Visual inspection of this figure demonstrates that middle-aged and older adults consistently perform more poorly across SOA for more recent items, with the exception of comparing the first two items presented. Thus, for four-item visual sequences, both middle-aged and older adults demonstrate a deficit in processing more recent items in the sequence.

#### Comparison of Auditory and Visual Results

Notable differences in performance are also apparent between these visual tasks and the auditory tasks measured in Experiment 1. All listener groups demonstrated a much more robust recency effect for auditory stimuli, consistent with a well-documented modality effect in which recent items are recalled better in auditory compared to visual presentations (Conrad and Hull, 1968). Crowder and Morton (1969) interpreted this effect as demonstrating that visual stimuli, in this case letters, are represented in a postcategorical memory store, while auditory presentations reflect precategorical and peripheral storage of auditory features (see also Frankish, 2008). Indeed, a reduced recency effect is also observed for categorically perceived stop consonants (e.g., Crowder, 1971). Thus, a strong recency effect for the vowel stimuli in this study may index the sustained presence of an early sensory memory trace that reflects continuous acoustic features of the stimulus (Pisoni, 1975).

The apparent difference in performance across sequence position suggests a possible dissociation between the perceptual abilities recruited for each task. It is notable that no significant correlations were obtained on performance between modalities for position accuracy. These results are consistent with recent reports of task and modality independence for sensory thresholds and gap detection with this same group of adults (Humes et al., 2009), although global sensory processing abilities may mediate age-related cognitive function (Humes et al., 2013). Overall, comparing results between visual and auditory modalities suggests sensory limitations in temporal processing as underlying age-related temporal processing decline. However, there does appear to be an amodal component, as older adults consistently perform poorer for later items in the sequence compared to their performance at other sequence positions. Thus, there may be a combination of sensory-specific limitations in processing and deficits with processing final items, possibly related to interference of earlier items in memory or difficulty in attentional components. Given the earlier theories underlying the recency effect (e.g., Crowder, 1971; Pisoni, 1975; Frankish, 1996), this may reflect poorer encoding or access to continuous, sensory memory stores by the older adults. Indeed, degrading the stimuli has a greater effect on the last item in auditory lists compared to other list items (Frankish, 1996; Surprenant, 1999).

## General Discussion

Overall, older adults as a group exhibit slower temporal processing for two- and four-item sequences in auditory and visual presentations compared to younger adults, with performance for the middle-aged adults falling in between the two groups.

For auditory two-item sequences, younger adults demonstrated better performance for identifying the second vowel in the sequence while older adults demonstrated no difference in recall for either sequence position. Overall, this pattern was fairly stable across the presentation rates tested. This may be consistent with holistic pattern perception at faster rates (Warren and Ackroff, 1976) or related to the reduced fine-grained stimulus representation and discriminability of final items in sensory memory (e.g., Surprenant and Neath, 1996). While younger and older performance was consistent across presentation rates, middle-aged adults demonstrated better identification for the second position with increasing rate.

For visual two-item sequences, older adults again demonstrated a different performance pattern. However, this time older adults identified the second item with greater accuracy and younger adults demonstrated no difference between the two sequence positions. This suggests the possible modality independence of age-related declines, arguing against a common source of processing deficits for this task (e.g., Lindenberger et al., 2001), and supports conclusions regarding different memory representations for auditory and visual stimuli (e.g., Crowder and Morton, 1969).

For the auditory four-item sequences, younger and middle-aged adults had nearly identical serial position curves at threshold. Remarkable similarity to these functions was apparent also for the older listener group. However, when examining the relative accuracy between sequential vowels, older adults consistently demonstrated poorer relative accuracy for final items compared to the other age groups. Thus, a combination of memory and temporal processing declines may contribute to the poorer performance for older adults on these tasks. However, the large similarity in performance at the other sequence positions suggests that temporal processing declines are responsible for the major differences in performance between the age groups.

Analysis of position accuracy for the visual four-item task revealed that all age groups had a marked absence of a recency effect, unlike what was found with auditory sequences. As discussed earlier, this modality effect is consistent with earlier reports of smaller recency effects for abstract, categorical processing of stimuli typically elicited for visual presentations (Crowder and Morton, 1969; Crowder, 1971; Pisoni, 1975; Frankish, 2008).

However, consistent across all of the tasks, except visual two-item sequences, was a deficit for older adults (and middle-aged adults for visual sequences) in processing later occurring items in a sequence. This pattern was most apparent on the state-trace analysis comparing item accuracy across the sequence. These results suggest that, particularly for longer or more complex tasks, older adults demonstrate more difficulty with final items regardless of modality. Investigations involving forward and backward masking in this same group of adults did not account for these differences in processing final items. Instead, the state-trace results may indicate that older adults have decreased sensory memory for the early, precategorical, continuous features of the stimuli (Pisoni, 1975; Frankish, 2008) and possibly a more degraded peripheral stimulus representation (Frankish, 1996; Surprenant, 1999).

With regard to the association between sensory and cognitive decline with age (Schneider and Pichora-Fuller, 2000), these results suggest that this interaction occurs, not only across global measures of sensory and cognitive abilities (Humes et al., 2013), but also operate within a single task, determining how an individual responds across trials. Humes et al. (2013) previously reported that these abilities are associated regardless of the age of the adult. The present analysis adds to this discussion by proposing that the relative influence of these abilities on task performance may change as a function of age—and ultimately determine individual performance and the type of errors that are made. Whether a causal uni- or bi-directional link exists between declines in temporal processing and amodal sensory memory function cannot be determined from the present analysis. In addition, while similar patterns are observed in auditory and visual modalities here and elsewhere (Visscher et al., 2007), it is not clear if a single sensory memory process or two sensory-specific memory processes are involved. Regardless of whether the neural architecture is shared, our results indicate similar processing is performed for longer (i.e., four-item) sequences and perhaps are susceptible to the same age-related physiological changes. However, it is clear that as adults age they may experience changes across the several different processing abilities that are required to complete any behavioral task, including psychophysical temporal order tasks. Understanding changes with age to the multicomponent processing of these tasks is important for identifying the source(s) of age-related changes in functioning.

Overall, older adults demonstrated highly similar performance to younger adults once accounting for declines in audibility and psychophysical processing of rapid temporal events. However, additional declines in performance were related to impaired sensory memory for recent items, particularly in more complex sequences. This is consistent with electrophysiological evidence demonstrating reduced sensory memory for auditory temporal processing in older adults for two-tone sequences (Rimmele et al., 2012). Functionally, this may mean that older adults have difficulty understanding speech not only due to the rapid transmission of acoustic features, but also because of poorer memory of fine-grained sensory detail. Poorer recall of the continuous acoustic features of speech may result in greater difficulties in degraded sensory environments (e.g., Surprenant and Neath, 1996; Surprenant, 1999, 2007) and result in poorer performance for tasks that rely on the fine-grained, non-categorical, detail of speech, particularly for vowels (Pisoni, 1975). The contribution of sensory-specific and amodal components parallels the dual memory components involved with auditory (i.e., speech) and visual (i.e., signed) language processing (Rönnberg et al., 2004). Thus, these results support previous reports from this dataset examining associations between different sensory and cognitive tasks (Humes et al., 2009, 2013), this time for examining multiple processes associated with item performance within a task: a combination of sensory-specific and sensory-general processes (e.g., short-term sensory memory) contribute to age-related declines in identifying rapid temporal events.

## Summary and Conclusions

Overall, large similarities were observed between the age groups for identification accuracy across rapid auditory and visual stimulus sequences when compared at equal performance levels. Thus, age-related temporal processing declines appear to be responsible for poorer identification thresholds among older adults. However, selective age group differences were observed for items in the final sequence position, with older adults demonstrating a final position identification deficit relative to the other groups for most tasks (auditory and visual) which may indicate a declining sensory memory store. Therefore, there are two factors that likely play a role in older adults’ serial order recall of rapid temporal sequences: sensory-specific processing that globally impacts task performance and an early sensory memory mechanism that contributes to decreasing performance for later items in rapid auditory and visual sequences, particularly for longer sequences.

## Author Contributions

LEH, TAB: conceptualization and methodological design of the experimental tasks; DF, LEH, TAB: data acquisition; DF, LEH: formulation of the manuscript research questions; DF, LEH, TAB: analysis and interpretation of the data; DF: writing the manuscript; and LEH, TAB: critical revision of the manuscript.

## Conflict of Interest Statement

The authors declare that the research was conducted in the absence of any commercial or financial relationships that could be construed as a potential conflict of interest.
